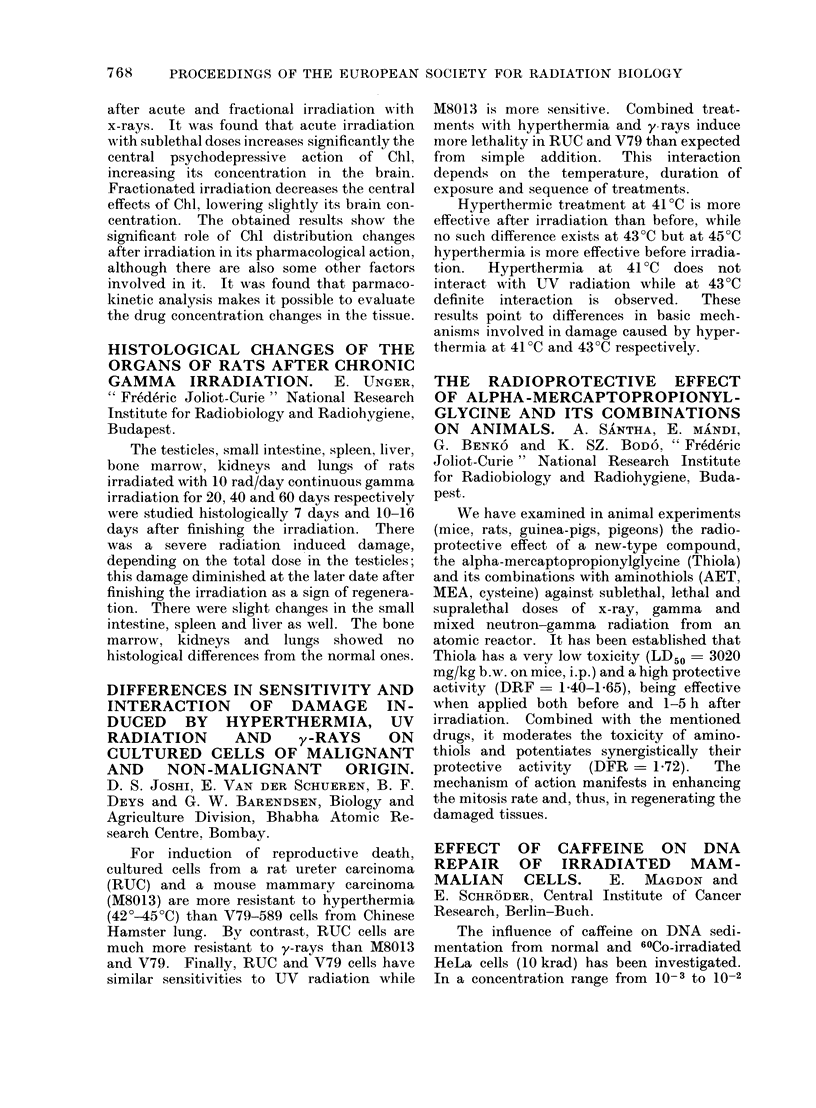# Proceedings: Histological changes of the organs of rats after chronic gamma irradiation.

**DOI:** 10.1038/bjc.1975.345

**Published:** 1975-12

**Authors:** E. Unger


**HISTOLOGICAL CHANGES OF THE ORGANS OF RATS AFTER CHRONIC GAMMA IRRADIATION.** E. Unger, " Fréderic Joliot-Curie " National Research Institute for Radiobiology and Radiohygiene, Budapest.

The testicles, small intestine, spleen, liver, bone marrow, kidneys and lungs of rats irradiated with 10 rad/day continuous gamma irradiation for 20, 40 and 60 days respectively were studied histologically 7 days and 10–16 days after finishing the irradiation. There was a severe radiation induced damage, depending on the total dose in the testicles; this damage diminished at the later date after finishing the irradiation as a sign of regeneration. There were slight changes in the small intestine, spleen and liver as well. The bone marrow, kidneys and lungs showed no histological differences from the normal ones.